# Aortic valve replacement in a 41-year-old woman with uncorrected tetralogy of Fallot, pulmonary atresia, and major aortopulmonary collateral arteries: a case report

**DOI:** 10.1186/s40981-023-00674-0

**Published:** 2023-12-06

**Authors:** Kazutomo Saito, Yudai Iwasaki, Takahiro Tasaki, Hidehisa Saito, Hiroaki Toyama, Yutaka Ejima, Masanori Yamauchi

**Affiliations:** 1 Anesthesiology and Perioperative Medicine, Tohoku University Graduate School of Medicine, 2-1 Seiryomachi, Aoba-Ku, Sendai, Miyagi 980-8575 Japan; 2 Department of Anesthesiology, Tohoku University Hospital, 1-1 Seiryomachi, Aoba-Ku, Sendai, Miyagi 980-8575 Japan; 3 Division of Surgical Center and Supply, Sterilization, Tohoku University Hospital, 1-1 Seiryomachi, Aoba-Ku, Sendai, Miyagi 980-8575 Japan

**Keywords:** Uncorrected tetralogy of Fallot, Major aortopulmonary collateral arteries, Infectious endocarditis, Aortic valve replacement

## Abstract

**Background:**

Tetralogy of Fallot (TOF) is a complex cyanotic congenital heart disease. As most patients with TOF undergo palliative or radical surgical repair during childhood, cardiac surgery under cardiopulmonary bypass (CPB) for adult survivors with unrepaired TOF is exceedingly rare.

**Case presentation:**

A 41-year-old woman with unrepaired TOF, pulmonary atresia (PA), and major aortopulmonary collateral arteries (MAPCAs) developed acute infectious endocarditis (IE). As vegetation gradually increased despite intravenous antibiotic administration, she was scheduled for urgent aortic valve replacement under CPB. Pulmonary blood flow was primarily provided by the MAPCAs originating from the descending aorta. Intra-aortic balloon occlusion for MAPCAs was performed to ensure a bloodless surgical field. Aortic valve replacement was successful.

**Conclusion:**

An adult with uncorrected TOF developed acute IE and subsequently had successful cardiac surgery under CPB. Understanding TOF physiology with PA and MAPCAs, particularly pulmonary blood flow through MAPCAs, is crucial.

## Background

Tetralogy of Fallot (TOF) is a complex cyanotic congenital heart disease characterized by ventricular septal defect (VSD), right ventricular outflow tract obstruction, overriding aorta, and right ventricular hypertrophy. Pulmonary atresia (PA) is an uncommon variant of TOF, and pulmonary circulation in patients with TOF and PA usually depends on the major aortopulmonary collateral arteries (MAPCAs). The combination of TOF with PA and MAPCAs is the most severe type of TOF [1].

Patients with TOF often undergo palliative or radical surgery during childhood. Without surgical treatment, approximately 3% of patients with TOF reach the fourth decade [2]. Some reports describe non-cardiac surgery for untreated TOF [3]; however, cardiac surgery under cardiopulmonary bypass (CPB) for adult survivors with unrepaired TOF is exceedingly rare [4]. The perioperative management of uncorrected TOF is challenging for anesthesiologists [5]. Herein, the case of a 41-year-old woman with uncorrected TOF with PA and MAPCAs who underwent aortic valve replacement (AVR) under CPB for active infectious endocarditis (IE) was presented.

## Case presentation

**Fig. 1:**
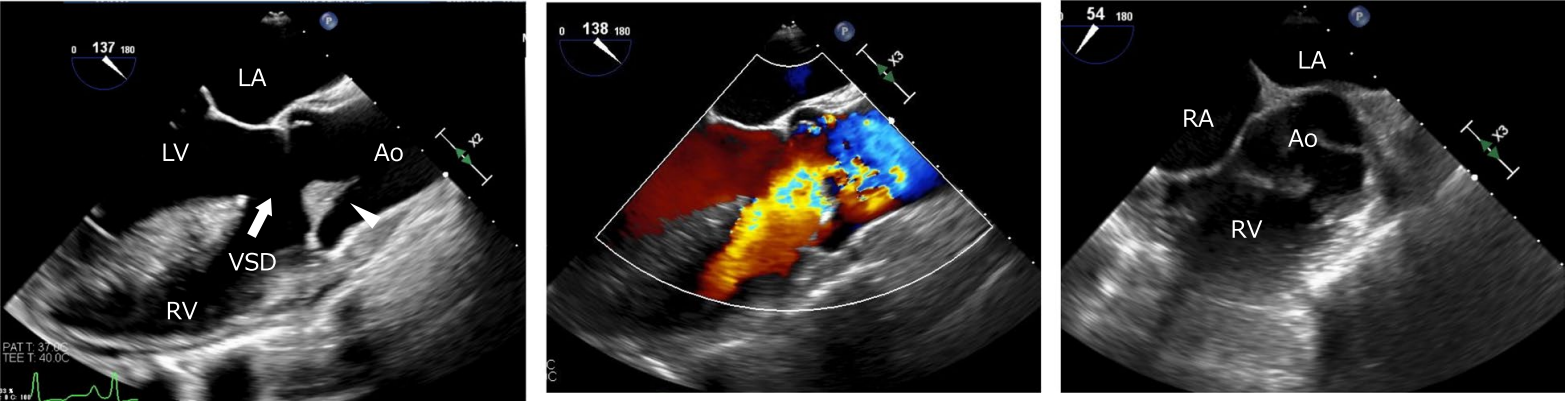
Transesophageal echocardiography revealed a 14 × 7 mm mobile vegetation attached to the right coronary cusp (arrowhead). Moderate aortic regurgitation was detected using transesophageal color Doppler imaging. The right ventricular outflow tract was not detected. Ao, aorta; LA, left atrium; LV, left ventricle; RV, right ventricle; VSD, ventricular septal defect

A 41-year-old woman (height 161.0 cm, body weight 45.4 kg) was transferred to our hospital because of acute IE and high fever. Blood cultures were positive for *Streptococcus pyogenes*. She was diagnosed with TOF accompanied by PA and MAPCAs in childhood. However, no radial surgery was performed because pulmonary blood flow was maintained through the MAPCAs. She was not taking any medications. Transesophageal echocardiography (TEE) performed at a local hospital revealed a 14 mm × 7 mm mobile vegetation attached to the right coronary cusp (Fig. 1), accompanied by moderate aortic regurgitation was also detected. The right ventricular outflow tract was not detected. There were no signs of heart failure. As vegetation gradually increased despite intravenous antibiotic administration, she was scheduled to undergo urgent AVR with CPB.

**Fig. 2:**
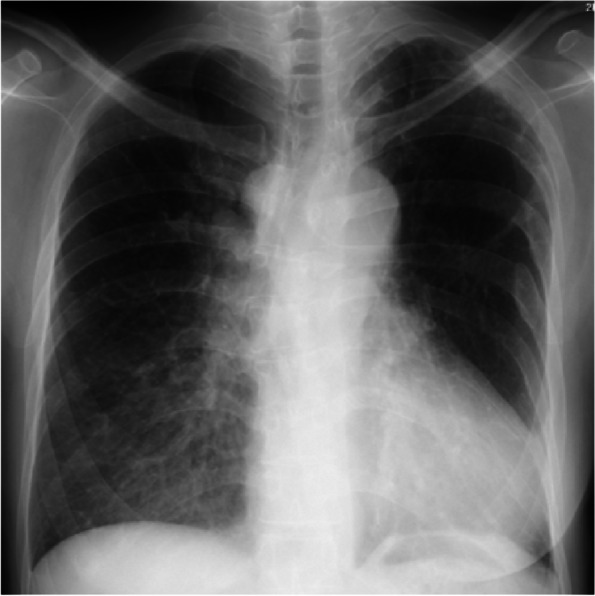
The anteroposterior chest radiography showed a boot-shaped heart and no pulmonary congestion

**Fig. 3:**
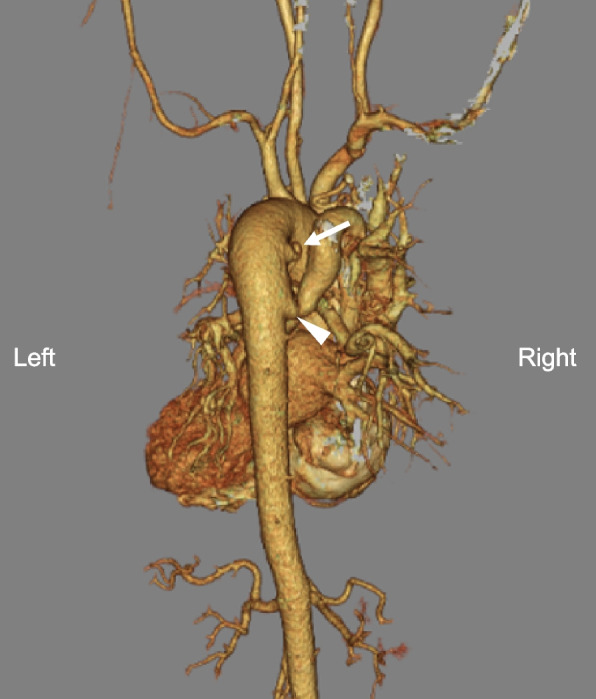
The 3D computed tomography shows that major aortopulmonary collateral arteries (MAPCAs) arise from descending aorta and course to bilateral pulmonary arteries. Upper MAPCA (arrow) courses to the left lung and the lower MAPCA (arrowhead) to the right lung

Upon admission, her oxygen saturation was 81% with 2 L/min via nasal cannula. Hemoglobin was elevated at 20.1 mg/dL (normal range: 12–16 mg/dL), and hematocrit was 61.6%. Chest radiography revealed a boot-shaped heart without pulmonary congestion (Fig. 2). Electrocardiography indicated sinus rhythm (heart rate 89/min) and right axis deviation. The 3D computed tomography shows that MAPCAs arise from descending aorta and course to bilateral pulmonary arteries (Fig. 3). Pulmonary blood flow through collateral flow returned directly to the left ventricle, venous drainage from only the SVC and IVC during CPB was predicted to be insufficient for adequate myocardial protection and a bloodless surgical field. Therefore, the intra-aortic balloon occlusion technique of the two main MAPCAs was planned.

In the operating room, the continuous monitoring included electrocardiogram, SpO_2_, arterial blood pressure, and arterial pressure-based cardiac output via a left radial artery catheter (FloTrac Sensor; Edwards Lifesciences Corporation, Irvine, CA, USA) before anesthesia induction. At baseline, NIBP was 126/74 mmHg, heart rate 102/min, and SpO_2_ 82% at 2 L/min via a nasal cannula. General anesthesia was induced by intravenous administration of midazolam (4 mg), 0.3 mg fentanyl, and 50 mg rocuronium. After tracheal intubation, a TEE probe was inserted. Additionally, a central venous catheter (PreSep Catheter; Edwards Lifesciences) was inserted via the right internal jugular vein to continuously monitor central venous pressure and oxygen saturation.

**Fig. 4:**
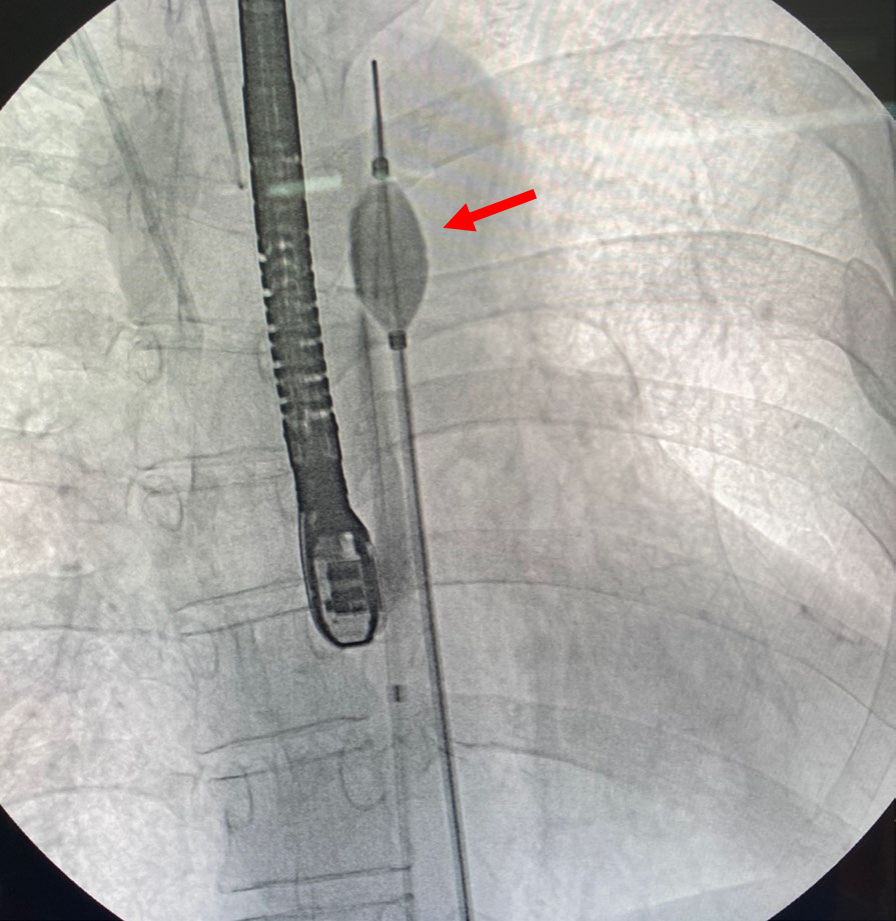
The fluoroscopy shows that the inflated intra-aortic balloon (arrow) was placed at the origin of the MAPCAs located in the descending aorta

**Fig. 5:**
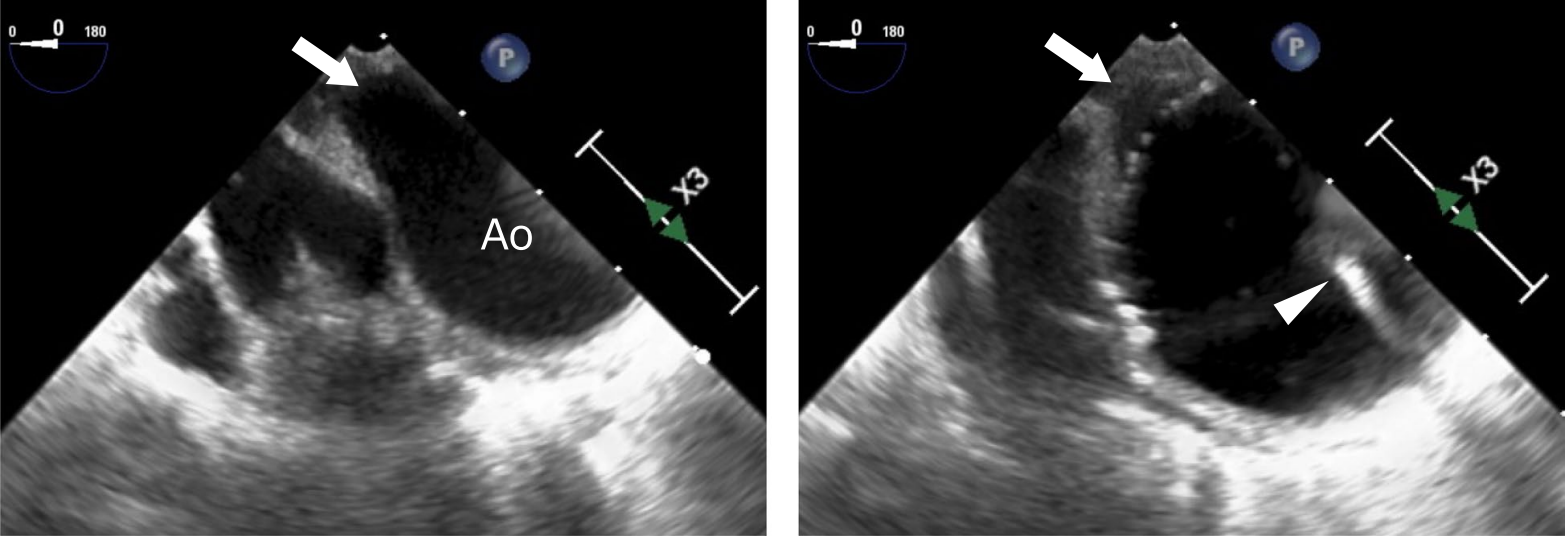
Transesophageal echocardiography revealed the MAPCA (arrow) originating from the descending aorta. The inflated intra-aortic balloon catheter (arrowhead) blocked the pulmonary blood flow through the MAPCA. Ao, aorta

Standard median sternotomy was performed under general anesthesia. The superior vena cava (SVC) and inferior vena cava (IVC) were used for venous drainage, and antegrade systemic perfusion was performed via the ascending aorta. As expected preoperatively, venous drainage from only the SVC and IVC was insufficient for adequate myocardial protection and a bloodless surgical field. Intra-aortic balloon via the femoral artery was inflated to block pulmonary blood flow through the two main MAPCAs. Balloon occlusion placement was confirmed using fluoroscopy (Fig. 4) and TEE (Fig. 5). It took about 15 min to place the intra-aortic balloon precisely. During balloon inflation, retrograde systemic perfusion was maintained via the femoral artery distal to the origin of the MAPCAs. A left atrial ventricular catheter was inserted. Myocardial protection was achieved using intermittent cold antegrade blood and retrograde cardioplegia.

A large VSD was directly observed under the right coronary cusp. In addition, vegetation was attached to the commissure between the noncoronary and right coronary cusp. During cardiac arrest, the patient’s aortic valve was replaced with a bioprosthetic valve (Inspiris®; Edwards Lifesciences Corporation). During CPB weaning, dobutamine infusion (2.0 µg/kg/min) was initiated to support single ventricle contraction. Balloon catheters were deflated and removed before CPB weaning, which was easily achieved. After weaning, SpO_2_ was maintained between 80 and 85%, similar to the preoperative value, and targeted a hemoglobin concentration of 15 mg/dL via red blood cell transfusion. Aortic cross-clamping, CPB, surgery, and anesthesia were performed for 115, 191, 520, and 631 min, respectively. The estimated blood loss and urine output were 4341 and 1400 mL, respectively. Overall, 2200 mL crystalloid, 1120 mL packed red blood cells, 960 mL fresh frozen plasma, 400 mL platelet concentrate, and 180 mL cryoprecipitate were administered.

After surgery, the patient was transferred to the intensive care unit with ventilator support under propofol sedation. The patient remained ventilated overnight and was extubated the following day. Four weeks later, the patient was discharged from the hospital without complications. Upon discharge, the oxygen saturation was 79% under oxygen administration (2 L/min via a nasal cannula).

## Discussion

TOF with PA and MAPCAs carries a 40% and 65% risk of death by 1 by 10 years of age, respectively, without surgery [6]. Only a few patients with TOF reach adulthood without surgical repair and only 3% survive into their fourth decade [2]. Consequently, cardiac surgery under CPB in adult patients with uncorrected TOF is exceedingly rare.

Successful aortic valve replacement owing to aortic valve stenosis or insufficiency is successfully performed after complete repair of TOF with PA and MAPCAs [7, 8]. However, to the best of our knowledge, only a few cases of aortic valve replacement for unrepaired TOF with PA and MAPCAs have been reported [9]. In this case, complete repair of the TOF with PA and MAPCAs was not an option; however, an open-heart surgical procedure with CPB was urgently required owing to the development of acute IE.

If the clinical situation gradually worsens despite intravenous antibiotic use for acute IE, emergency surgical intervention may be beneficial, even for adult patients with uncorrected TOF. In normal cardiac surgery, venous drainage from both the SVC and the IVC during CPB is sufficient to ensure a bloodless surgical field. However, if pulmonary blood flow relies primarily on MAPCAs originating from the descending aorta, as observed here, SVC and IVC drainage alone is insufficient. Therefore, pulmonary blood flow through the MAPCAs was blocked using the intra-aortic balloon occlusion technique. For safe cardiac surgery, it is essential to control collateral flow in patients with TOF/PA/MAPCA.

In conclusion, an adult patient with uncorrected TOF with PA and MAPCAs developed acute IE and underwent cardiac surgery under CPB without any complications. To date, there have only been a few reports of AVR in older patients with previously uncorrected TOF. Understanding the physiology of TOF with PA and MAPCAs is important, especially regarding pulmonary blood flow through MAPCAs. If systemic and pulmonary blood flows exit through a single great artery, the establishment of a CPB becomes complicated.

## Abbreviations

TOF: Tetralogy of Fallot
VSD: Ventricular septal defect
PA: Pulmonary atresia
MAPCAs: Major aortopulmonary collateral arteries
CPB: Cardiopulmonary bypass
AVR: Aortic valve replacement
IE: Infectious endocarditis
TEE: Transesophageal echocardiography
SVC: Superior vena cava
IVC: Inferior vena cava
